# The impacts of the “4+7” pilot policy on the volume, expenditures, and daily cost of Serotonin-Specific Reuptake Inhibitors (SSRIs) antidepressants: A quasi-experimental study

**DOI:** 10.3389/fphar.2022.829660

**Published:** 2022-08-17

**Authors:** Xiaotong Wen, Zhaolun Wang, Luxinyi Xu, Jia Luo, Xin Geng, Xiaoze Chen, Ying Yang, Dan Cui, Zongfu Mao

**Affiliations:** ^1^ School of Public Health, Wuhan University, Wuhan, China; ^2^ Global Health Institute, Wuhan University, Wuhan, China; ^3^ Xi’an Jiao Tong Liverpool University, Suzhou, China

**Keywords:** the “4+7” pilot policy, volume-based procurement, price, expenditures, serotonin-specific reuptake inhibitors (SSRIs), antidepressants, interrupted time-series analysis (ITSA), quasi-experimental design and analysis

## Abstract

**Objectives:** The purpose of this study was to quantitatively evaluate the impacts of the”4 + 7” pilot policy on purchase volume, purchase expenditures, and daily cost and to find the changes in the use of SSRIs.

**Methods:** Data was collected covering 31 months, before, during, and after the “4 + 7” pilot policy was implemented in Shenzhen. Interrupted time-series (ITS) analysis was used to examine whether there had been a significant effect with the onset of the “4 + 7” pilot policy in March 2019.

**Findings:** The daily cost of policy-related drugs had a substantial drop of 2.93 yuan under the “4 + 7” pilot policy. The result has shown a 76.70% increase in volume and a 3.39% decrease in the expenditure on policy-related drugs. This study found that the “4 + 7” pilot policy increased the proportion of purchasing winning drugs, with an increment of 85.60 percent. After the implementation of the “4 + 7” pilot policy, policy-related drugs decreased by 443.55thousand Chinese yuan. The study indicated that volume of winning products significantly increased as shown in the regression with a level coefficient (*β*_*2*_) of -224.17 (*p* < 0.001) and trend coefficient (*β*_*3*_) of 15.74 (*p* < 0.001). The result revealed that both volume and expenditures on branded products showed a significant decrease in the regression in the post-intervention period (level coefficient of volume: *β*_*2*_ = -57.65, *p* < 0.01, trend coefficient of volume: *β*_*3*_ = -3.44, *p* < 0.01; level coefficient of expenditure: *β*_*2*_ = -712.98, *p* < 0.01, trend coefficient of expenditure: *β*_*3*_ = -40.10, *p* < 0.01).

**Conclusion:** The volume-based procurement has successfully led to price reductions and improved the affordability of medicines, especially for those with chronic diseases. The volume-based procurement has demonstrated initial success in reshaping the composition of the Chinese pharmaceutical market in favor of generics with high quality and low prices.

## Introduction

Global drug costs are growing rapidly and are set to exceed $1.5 trillion by 2023 (Science HD, 2019). Growing pharmaceutical spending remains a persistent challenge in many countries all over the world. Large sections of the global population can’t afford pharmaceutical spending (Cameron et al., 2009; Babar et al., 2019; Rodwin, 2021; San-Juan-Rodriguez et al., 2021). Notably, the problem is more severe in low-middle-income countries. Pharmaceutical expenditure in lower-middle-income countries can be up to 70% of total health expenditure, compared with 17% in higher-income countries (Papanicolas et al., 2018; Parente, 2018). China’s economic burden on pharmaceuticals has increased steadily over the last decade. Pharmaceutical spending doubled from 2009 to 2017, reaching up to 34% of total health care expenditure in 2017 (China National Health Development Research Center, 2018). The percentage of pharmaceutical expenses in total health care expenditure in China was much higher than in some developed countries such as the United States (12.6%) and Australia (13.8%), as well as most Asian countries such as Japan (17.8%) and South Korea (19.3%) (OECD, 2021). China’s relatively high proportion of drug costs has induced an increasing financial burden on patients. Thus, the Chinese government is currently exploring strategies to contain rapidly growing pharmaceutical spending.

Pharmaceutical expenditures depend on drug prices and drug volume, which were corresponding to the supply and demand sides of drugs respectively (Han et al., 2015). Price reduction strategies were direct measures to reduce pharmaceutical expenditures, which were associated with the supply side of drugs (Hakonsen et al., 2009; Lee et al., 2015; Rodwin, 2020; Yousefi et al., 2020). In China, to lower the prices of procured drugs, the Volume-Based Drug Centralized Procurement National Pilot Policy was officially launched in March 2019, aiming to reduce intermediates and marketing costs, promote marketing mode adjustment, and purify industry ecology. Four municipalities (Beijing, Tianjin, Shanghai, and Chongqing) and seven sub-provincial cities (Shenyang, Dalian, Xiamen, Guangzhou, Shenzhen, Chengdu, and Xi’an) were chosen as pilot cities. Therefore, the Volume-Based Drug Centralized Procurement Pilot Policy is also called the “4 + 7” pilot policy.

In the “4 + 7″ pilot policy, the purchase volume was pre-defined by centralizing the purchase volume from the public medical institutions in 11 selected cities. The Volume-Based Drug Centralized Procurement aimed to achieve lower prices through large-volume procurement, to implement the so-called “volume for price” strategy. It also can be seen as group purchasing which had bargaining power in the drug purchasing process (Noto et al., 2017). The drug supply enterprises reduced drug prices to obtain a larger market. Only one company would win the bidding for each policy-related drug, and the purchasing cycle was 12 months. The drug will be purchased with a bidding price until the purchase cycle expired. The policy-related drugs include branded drugs, generic drugs, and corresponding reference preparations. The quality of policy-related drugs was ensured by Generic Quality Consistency Evaluation (GQCE) approval. The prices of policy-related drugs were chopped, and price cuts ranged from 25 to 96% (Yuan et al., 2021). The massive price cuts dramatically impacted overall drug expenditures.

Depression is characterized by marked and lasting depressed mood and sadness, slow thinking, loss of interest or pleasure, decreased willpower, low self-worth, feelings of tiredness, and poor concentration (Guajardo et al., 2013; Lim et al., 2018). Depression is the leading cause of suicide in China (Phillips et al., 2002; Cheng et al., 2020). In the most severe form of depression, it can lead to suicide and increased risk of mortality (Yang et al., 2013). Furthermore, patients experiencing depression may endure periodic irritation, anxiety, emotional disorders, and/or other mental agonies (Jantaratnotai et al., 2017); therefore, taking the antidepressant for the patients is a relatively long even lasting process. As one of the most common mental disorders, depression is characterized by a significant and continuous low mood state and seriously affects the patients’ learning ability as well as life and social functions (WHO, 2017).

In China, with the rapid economic development, the accelerated pace of modern life, and the increasingly fierce social competition, life pressure is also increasing, and depression has become a common public health problem. With 50 million depression patients in China, the DALYs have increased by 36.5% in the past 30 years (Ren et al., 2020). If appropriate treatment is not applied, patients may develop a disability, premature death, and severe aftermath to their families from depression. However, in a national cross-sectional epidemiological survey from 157 representative points in 31 provinces across China, only 0.5% of participants with depressive disorders were treated adequately (Yu et al., 2021), indicating that only a few people have received adequate treatments. If the treatments of depression are not effective and standardized, it will result in a huge social and economic burden. A previous survey estimated the economic consequences of depressive disorders in China, conducted in five cities (Beijing, Changsha, Chengdu, Shanghai, and Suzhou) which represented the four broad geographic areas in China (North, Central, Southwest, and East Coast regions). As per the result of this survey, the proportion of medication costs in outpatients was 74.01% (Hu et al., 2007). To a certain extent, reducing the burden of drug costs can improve the compliance of depressive disorder patients. In China, national programs are needed to remove barriers to accessibility, availability, and affordability of medication treatment for depression (Yu et al., 2021).

After the “4 + 7″ pilot policy, more patients could receive drug treatments. Considering the incidence of depression in the Chinese population and the economic burden of the disease, this study limits the research scenario to Serotonin-Specific Reuptake Inhibitors (SSRIs) drugs, which are the most recommended treatments for depression according to the second version of the Chinese Guideline for Prevention and Treatment of Depression (Ya-June 2018).

The purpose of this study was to quantitatively evaluate the impacts of the “4 + 7” pilot policy on purchase volume, purchase expenditures, and daily cost and to find the changes in the use of SSRIs. Firstly, this study aimed to verify whether the “4 + 7” pilot policy would lead to a decrease in the overall expenditure of SSRIs. Second, the research team tried to conduct a subgroup analysis for policy-related drugs and alternative drugs, winning drugs and non-winning drugs, generic drugs, and branded drugs. Last but not least, our team members examined the trend of the volumes of and the expenditures on SSRIs and their subgroups.

## Materials and methods

### Data sources

Data on products purchased between June 2017 and December 2019 were extracted from the Drug Trading Platform of Shenzhen—Shenzhen Group Purchasing Organization (Shenzhen GPO). The team was able to compare the changes in volume, expenditure, and daily costs of drugs for depression treatment after a national-level interference. The project collected monthly drug purchase orders from June 2017 to December 2019 in each medical institution. Each drug purchase order included the code of drug, generic name, dosage form, strengths, procurement unit, price per unit, net quantity of contents, pharmaceutical manufacturer, medical institution, purchase date, purchase volume, purchase expenditures, etc.

### Study setting

As the first of China’s Special Economic Zones, Shenzhen has undergone unprecedented economic development and social change, which has also led to tremendous changes in disease epidemiology (Gong et al., 2012). Consequently, the prevalence of depressive disorders in Shenzhen was the highest in China (Searle et al., 2019). Shenzhen started to investigate group purchasing strategies for certain types of drugs such as before and became one of 11 pilot cities to carry out drug volume-based purchasing. In this study, the team analyzed the SSRIs class which included Escitalopram, Paroxetine, Citalopram, Fluvoxamine, Fluoxetine, and Sherqulin. Escitalopram and Paroxetine were policy-related drugs. Citalopram, Fluvoxamine, Fluoxetine, and Sherqulin were alternative drugs. Then the research team divided policy-related drugs (including Escitalopram and Paroxetine) into two groups (winning products and non-winning products) based on whether the drugs won the bid in the “4 + 7” pilot policy. Only one company would win the bidding for each policy-related drug. Policy-related drugs were also divided into two subgroups based on whether the drugs were branded drugs or generic drugs. In this article, “product” hereafter designates a distinctive strength of a drug produced by a particular pharmaceutical manufacturer under the same generic name or the same strengths produced by different pharmaceutical manufacturers under the same generic name.

### Variables and measurements

The primary measure is aimed at purchase volume, purchase expenditures, and daily costs of drugs. The defined daily dose (DDD) is the average daily dose of a particular drug set for use in adults for the treatment of a primary indication. The DDD of Escitalopram, Paroxetine, Citalopram, Fluvoxamine, Fluoxetine, and Sherqulin were 10, 20, 20, 100, 20, and 50 mg respectively. DDDs were standard measurements to calculate and compare drug purchase volume. According to the WHO Collaborating Centre for Drug Statistics Methodology (WHO, 2021), DDDs were calculated by the formula:

$${\text{DDD}}_{\text{s}}=\overset{n}{\underset{i=1}{\sum }}\left(\frac{\text{net quantity of contents}\times \text{strengths}}{DDD}\times N\right)$$

In this formula, the net quantity of contents expresses the numerical count of one specific drug in a marketed inner retail container (usually interchangeably immediate container in China). The net quantity of contents is the number of units of preparation contained in the smallest sales packaging unit. The strength is the amount of drug in the dosage form or a unit of the dosage form (e.g. 10 mg capsule, 20 mg/5 ml suspension). Thus, this formula can conduct calculations (in this case, additions or subtractions) between different products with different net quantities of contents and/or different strengths of drugs with a standardized and unified measurement that multiplied “net quantity of contents” and “strengths”, divided by “Defined Daily Doses,” and finally timed by the total purchased quantity. Each drug purchase order can be measured by DDDs. Then we convert the purchase volume of drugs to DDDs which was a standardized and unified consumption unit for this situation (Wessling and Boethius, 1990; Rodriguez and Vega, 2010). DDDs used as the drug utilization index comparable across regions, countries, and stages, allowing for long-term monitoring and continuous evaluation of drug utilization (Natsch et al., 1998).

The purchase expenditures were calculated by the amount of drug purchase orders in Chinese yuan (CNY). The daily costs of drugs were measured by Defined Daily Dose cost (DDD), a standard measure of the procurement cost of each product (Guan et al., 2018). In this study, DDDc was calculated by the ratio of expenditures and DDDs.

### Statistical analysis

Two types of analysis were applied in this study: descriptive analysis and interrupted time-series analysis (ITSA). Descriptive analysis was used to present differences in DDDs, expenditures, and DDDc of SSRIs between before and after implementation of the “4 + 7” pilot, as the policy was effective in March 2019.

The effect of the “4 + 7” pilot policy was evaluated by interrupted time-series (ITSA) with segmented regressions. ITS was the best and most commonly used approach for evaluating the longitudinal effects on interventions occurring at a fixed point of time, e.g. the date on which the policy was implemented (Xiao et al., 2021). Many researchers considered ITS analysis as the most practical quasi-experimental design to evaluate the effects of interventions (Zhao et al., 2021). The model this study uses a linear trend in the outcome within each segment. The specification of the linear regression model to be analyzed is as the following equation:

$${\text{Y}}_{\text{it}}=\beta_{0}+\beta_{1}{\text{∗Time}}_{\text{t}}+\beta_{2}{\text{∗Intervention}}_{\text{t}}+\beta_{3}\text{∗Time}\_\text{after}\_\text{Intervention}+\beta_{4}\text{∗sin}\left(2\pi m_{i}\right)+\beta_{5}\text{∗cos}\left(2\pi m_{i}\right)+\varepsilon_{\text{it}}$$

In this model, Y_it_ is the independent outcome variable (DDDs, expenditures, or DDDc). *β*_*0*_ reflects the baseline level of the outcome, which is a constant. *β*_1_ represents the change in the baseline trend that is independent of the intervention, which is the structural trend. *β*_2_ captures the change in the level of the outcome, representing SSRIs use after the intervention; and *β*_3_ estimates the change in trend in SSRIs use after the intervention. Some previous studies revealed a seasonal trend of depression (Yang et al., 2010; Ayers et al., 2013; Soreni et al., 2019). In this study, the seasonality effect was considered by *β*_4_ and *β*_5._ $m_{i}$ is equal 1/12 for January, 2/12 for February, and so on with 12/12 for December (Hunsberger et al., 2002). *ε*_it_ is an estimate of the random error at Time_t_ (Lagarde, 2012). In this research, ITS was utilized to evaluate the impact on DDDs, expenditures, and DDDc after the implementation of the policy. The time of implementation of the “4 + 7” pilot policy in March 2019 was regarded as the intervention time point for ITS analysis. Intervention—binary indicator denoting 0 during the pre-intervention period and one during the postintervention period. Time since intervention (in months)—ordinal indicator denoting months since time interruption (i.e., implementation of intervention). Therefore, two segments with one interruptive point were constructed, where one is the pre-intervention period (from June 2017 to February 2019), and the other one is the post-intervention period (from March 2019 to December 2019).

Pre-requisite tests were conducted, for example, unit roots, white noise test, and autocorrelation (Phillips and Perron, 1988; Goodhart et al., 1993). Autocorrelation may lead to underestimated standard errors and overestimated significance of the effects of an intervention. Durbin–Watson statistic was performed to ensure that models adequately corrected for first-order autocorrelation. Values of the Durbin–Watson statistic close to 2.0 indicated the absence of serial autocorrelation (Bohnert et al., 2018). The specific estimation method in the ITS analysis was Newey-West standard errors for coefficients estimated. If autocorrelation is detected, a generalized least squares estimator, such as the Prais–Winsten method, was used to estimate the regression (Wagner et al., 2002; Lagarde, 2012). Data management and analysis were performed using Stata 16.0 (Stata Corporation, College Station, TX, United States). Statistical significance was noted when *p*-values were less than 0.05.

## Results

### Descriptive analysis of changes in volume, expenditures, and DDDc

Six SSRIs (Escitalopram, Paroxetine, Citalopram, Fluvoxamine, Fluoxetine, Sherqulin) were included in this study where Escitalopram and Paroxetine were “4 + 7″ policy-related drugs and their alternatives were Citalopram, Fluvoxamine, Fluoxetine and Sherqulin (Table 1). The applied method of analysis was descriptive statistics, which was designed to compare the measures from two periods, which were 10 same selected months (March to December) from 2018 to 2019, since the intervention of the policy was launched in March 2019, and this study sought to conduct an unbiased comparison between pre-and post-intervention of the policy. Descriptive statistics also calculated the growth rate between the two periods. In Table 2, the DDDs indicated that the purchase volume of Escitalopram and Paroxetine increased by 76.70% after the intervention of the “4 + 7” policy; meanwhile, the expenditures and DDDc of Escitalopram and Paroxetine drugs decreased by 3.39%, 45.32% respectively. The DDDs and expenditures on alternative drugs increased by 26.71%, and 26.66% respectively. The DDDs and expenditures of SSRIs increased by 49.85%, and 11.20% respectively. The DDDc of SSRIs decreased by 25.79%. As shown in Supplementary Figures S1, 2 , the DDDs and expenditures of SSRIs increased after the policy interv*ention. At the same time, the DDDc of SSRIs and policy-related drugs both were decrease*d (SupplementaryFigure S3)*.*

**TABLE 1:** The information of all the SSRI medicines.

Category	Winning/Non-winning	Branded/Generic	DDD (mg)	Number of products	Number of pharmaceutical manufacturers
Policy-related drugs					
Escitalopram	Winning products	Generic drugs	10	1	1
Escitalopram	Non-winning products	Branded drugs	10	2	2
Escitalopram	Non-winning products	Generic drugs	10	2	2
Paroxetine	Winning products	Generic drugs	20	1	1
Paroxetine	Non-winning products	Branded drugs	20	1	1
Paroxetine	Non-winning products	Generic drugs	20	1	1
Alternative drugs					
Citalopram	-	-	20	0	0
Fluvoxamine	-	-	100	0	0
Fluoxetine	-	-	20	2	2
Sherqulin	-	-	50	2	2

**TABLE 2:** Descriptive analysis of SSRIs in Shenzhen.

Categories	DDDs (thousand)			Expenditures (thousand CNY)			DDDc (CNY)		
	Mar.-December 2018	Mar.-December 2019	Growth rate (%)	Mar.-December 2018	Mar.-December 2019	Growth rate (%)	Mar.-December 2018	Mar.-December 2019	Growth rate (%)
Policy-related drugs	1,924.99	3,401.49	76.70	13,100.32	12,656.86	-3.39	6.81	3.72	-45.32
Escitalopram	1,045.15	1,691.52	61.85	9,152.14	9,283.74	1.44	8.76	5.49	-37.32
Paroxetine	879.84	1,709.97	94.35	3,948.18	3,373.12	-14.57	4.49	1.97	-56.04
Alternative drugs	2,233.48	2,830.06	26.71	12,366.51	15,662.93	26.66	5.54	5.53	-0.04
Fluoxetine	467.49	623.28	33.33	3,589.83	4,710.64	31.22	7.68	7.56	-1.58
Sherqulin	1,765.99	2,206.78	24.96	8,776.68	10,952.30	24.79	4.97	4.96	-0.14
SSRI	4,158.46	6,231.55	49.85	25,466.83	28,319.79	11.20	6.12	4.54	-25.79

*The DDDs and expenditures of Escitalopram increased by 61.8*5%, and 1.44%, respectively; the DDDc of Escitalopram decreased by 37.32%. The DDDs of Paroxetine increased by 94.35%; the expenditures and DDDc of Paroxetine decreased by 14.57%, and 56.04%, respectively. The DDDs of Fluoxetine and Sherqulin increased by 33.33%, and 24.96% respectively.

The constituent ratio (CR) of DDDs and expenditures of SSRIs between pre-and post-intervention periods are listed in Table 3. Before implementation of the policy, 25.13% of Escitalopram and 21.16% of Paroxetine comprised SSRIs measured in DDDs, while DDDs of both Escitalopram and Paroxetine increased after the policy was implemented. Especially, DDDs of Paroxetine increased by 6.28 percent. DDDs of policy-related drugs increased by 8.29 percent in the post-intervention period, and expenditures on policy-related drugs dropped by 6.75 percent. Moreover, the DDDs of Sherqulin decreased by 7.05 percentage points, while expenditures raised by 4.21 percentage points with the interference of the “4 + 7” policy.

**TABLE 3:** Descriptive analysis of SSRIs CR in Shenzhen.

Categories	The CR of DDDs (%)			The CR of expenditures (%)		
	Mar.-December 2018	Mar.-December 2019	Variation	Mar.-December 2018	Mar.-December 2019	Variation
**Policy-related drugs**	46.29	54.58	8.29	51.44	44.69	-6.75
Escitalopram	25.13	27.14	2.01	35.94	32.78	-3.16
Paroxetine	21.16	27.44	6.28	15.50	11.91	-3.59
**Alternative drugs**	53.71	45.42	-8.29	48.56	55.31	6.75
Fluoxetine	11.24	10.00	-1.24	14.10	16.63	2.54
Sherqulin	42.47	35.41	-7.05	34.46	38.67	4.21
**SSRI**	100.00	100.00	0.00	100.00	100.00	0.00

As displayed in Table 4, the DDDs and expenditures of all products in the non-winning group decreased by 74.55%, and 68.02%, respectively. The DDDc of policy-related products in the non-winning group increased by 25.64%. Table 4 proved that the DDDs of Escitalopram in the winning group increased from 0 to 1311.11 thousand. The DDDc of Entecavir, as a winning product, was 4.42 CNY. The DDDs of Paroxetine, another winning product, increased from 0 to 1600.44 thousand, and the DDDc of it was 1.67 CNY. Supplementary Figures S4, 5 revealed that the DDDs and expenditures of winning products were increased which both were 0 before the implementation of the “4 + 7” policy. Supplementary Figure S6 revealed that the DDDc of policy-related drugs decreased.

**TABLE 4:** Descriptive analysis of SSRI policy-related drugs in Shenzhen.

Categories	DDDs (thousand)			Expenditures (thousand CNY)			DDDc (CNY)		
	Mar.-December 2018	Mar.-December 2019	Growth rate (%)	Mar.-December 2018	Mar.-December 2019	Growth rate (%)	Mar.-December 2018	Mar.-December 2019	Growth rate (%)
**Policy-related drugs**	1,924.99	3,401.49	76.70	13,100.32	12,656.86	-3.39	6.81	3.72	-45.32
Winning products	0.00	2,911.55	-	0.00	8,467.86	-	-	2.91	-
Non-winning products	1,924.99	489.94	-74.55	13,100.32	4,189.00	-68.02	6.81	8.55	25.64
Escitalopram	1,045.15	1,691.52	61.85	9,152.14	9,283.74	1.44	8.76	5.49	-37.33
Winning products	0.00	1,311.11	-	0.00	5,795.12	-	-	4.42	-
Non-winning products	1,045.15	380.41	-63.60	9,152.14	3,488.62	-61.88	8.76	9.17	4.73
Paroxetine	879.84	1,709.97	94.35	3,948.18	3,373.12	-14.57	4.49	1.97	-56.04
Winning products	0.00	1,600.44	-	0.00	2,672.74	-	-	1.67	-
Non-winning products	879.84	109.53	-87.55	3,948.18	700.38	-82.26	4.49	6.39	42.50
**Policy-related drugs**	1,924.99	3,401.49	76.70	13,100.32	12,656.86	-3.39	6.81	3.72	-45.32
Branded drugs	801.24	342.59	-57.24	9,113.00	3,595.24	-60.55	11.37	10.49	-7.73
Generic drugs	1,123.75	3,058.90	172.21	3,987.32	9,061.61	127.26	3.55	2.96	-16.51
Escitalopram	1,045.15	1,691.52	61.85	9,152.14	9,283.74	1.44	8.76	5.49	-37.32
Branded drugs	549.74	252.66	-54.04	7,080.63	2,954.62	-58.27	12.88	11.69	-9.21
Generic drugs	495.41	1,438.86	190.44	2,071.52	6,329.12	205.53	4.18	4.40	5.20
Paroxetine	879.84	1,709.97	94.35	3,948.18	3,373.12	-14.57	4.49	1.97	-56.04
Branded drugs	251.50	89.93	-64.24	2,032.37	640.62	-68.48	8.08	7.12	-11.85
Generic drugs	628.34	1,620.04	157.83	1,915.81	2,732.50	42.63	3.05	1.69	-44.68

<On the other hand, for those policy-related generic products, Table 4 indicated that the DDDs and the expenditures of them increased by 172.21%, and 127.26%, respectively, while the DDDc of all policy-related generic products decreased by 16.51%. Moreover, DDDs of generics of Escitalopram increased by 190.44%; meanwhile, expenditures and DDDc of those generics of Escitalopram also increased by 205.53 and 5.20%, respectively.

Supplementary Figures S7, 8 revealed that the DDDs and expenditures of branded drugs were obviously decreased after the implementation of the “4 + 7” policy. Supplementary Figure S9 revealed that the DDDc of policy-related drugs decreased.

Table 5 demonstrated the CR changes of DDDs, expenditures, and DDDc of winning and non-winning products, branded and generic products between pre-and post-intervention periods. Before the implementation of the “4 + 7” pilot policy, in public hospitals, the market share was 0 for some specific products of Escitalopram and Paroxetine in the winning group but after the implementation of the policy, these products of Escitalopram and Paroxetine achieved significant growth with market shares of 38.55 and 47.05%, respectively. The market share of policy-related generic products was 58.38% before intervention. Finally, the market share of Escitalopram and Paroxetine generic products in SSRIs measured by DDDs increased by 16.57 and 14.99%, respectively.

**TABLE 5:** Descriptive analysis of SSRIs policy-related CR drugs in Shenzhen.

Categories	The CR of DDDs (%)^a^			The CR of expenditures (%)^a^		
	Mar.-December 2018	Mar.-December 2019	Variation	Mar.-December 2018	Mar.-December 2019	Variation
Policy-related drugs	100.00	100.00	0.00	100.00	100.00	0.00
Winning products	0.00	85.60	85.60	0.00	66.90	66.90
Non-winning products	100.00	14.40	-85.60	100.00	33.10	-66.90
Escitalopram	54.29	49.73	-4.56	69.86	73.35	3.49
Winning products	0.00	38.55	38.55	0.00	45.79	45.79
Non-winning products	54.29	11.18	-43.11	69.86	27.56	-42.30
Paroxetine	45.71	50.27	4.56	30.14	26.65	-3.49
Winning products	0.00	47.05	47.05	0.00	21.12	21.12
Non-winning products	45.71	3.22	-42.49	30.14	5.53	-24.60
Policy-related drugs	100.00	100.00	0.00	100.00	100.00	0.00
Branded drugs	41.62	10.07	-31.55	69.56	28.41	-41.16
Generic drugs	58.38	89.93	31.55	30.44	71.59	41.16
Escitalopram	54.29	49.73	-4.56	69.86	73.35	3.49
Branded drugs	28.56	7.43	-21.13	54.05	23.34	-30.71
Generic drugs	25.74	42.30	16.57	15.81	50.01	34.19
Paroxetine	45.71	50.27	4.56	30.14	26.65	-3.49
Branded drugs	13.07	2.64	-10.43	15.51	5.06	-10.45
Generic drugs	32.64	47.63	14.99	14.62	21.59	6.96

a The CR, presented in Table 5 shows only the proportion of all policy-related drugs.

### ITS analysis of changes in DDDs, expenditures, and DDDc

Table 6 represented the results of the segmented linear analysis with ITS. The DDDc of SSRIs dropped by 1.56 yuan (*p* < 0.001) with the “4 + 7” pilot policy.

**TABLE 6:** The result of the ITS analysis of SSRIs in Shenzhen.

Categories	DDDs (thousands)	Expenditures (thousands of CNY)	DDDc (CNY)
	Coef. (95%*C.I.*)	Coef. (95%*C.I.*)	Coef. (95%*C.I.*)
Policy-related drugs			
Baseline trend *β*_*1*_	5.41 (0.83,9.98)*	36.81 (8.59,65.02)*	-0.01 (-0.07,0.05)
Change in level *β*_*2*_	67.34 (-1.41,136.09)	-443.55 (-865.17,-21.93)*	-2.93 (-3.68,-2.19)***
Change in trend *β*_*3*_	4.93 (-6.73,16.58)	-3.64 (-40.25,32.97)	-0.02 (-0.16,0.11)
Seasonal effects sin *β*_*4*_	-1.01 (-37.21,35.20)	36.68 (-190.33,263.70)	0.10 (-0.27,0.47)
Seasonal effects cos *β*_*5*_	-10.20 (-41.68,21.27)	-65.62 (-219.55,88.30)	-0.27 (-0.60,0.06)
Constant *β*_*0*_	114.14 (74.72,153.56)***	797.39 (575.43,1019.35)	7.15 (6.42,7.88)***
Escitalopram			
Baseline trend *β*_*1*_	2.88 (0.31,5.45)*	27.70 (8.22,47.17)**	0.04 (-0.06,0.14)
Change in level *β*_*2*_	5.50 (-28.60,39.60)	-350.09 (-653.31,-46.86)*	-3.17 (-4.33,-2.01)***
Change in trend *β*_*3*_	6.06 (0.89,11.22)*	9.50 (-13.56,32.56)	-0.16 (-0.39,0.07)
Seasonal effects sin *β*_*4*_	4.15 (-17.12,25.42)	44.22 (-126.40,214.84)	-0.07 (-0.64,0.50)
Seasonal effects cos *β*_*5*_	-8.97 (-25.10,7.17)	-70.32 (-185.50,44.86)	-0.16 (-0.54,0.23)
Constant *β*_*0*_	64.28 (44.09,84.47)*	540.59 (402.26,678.93)***	8.50 (7.46,9.55)***
Paroxetine			
Baseline trend *β*_*1*_	2.52 (0.30,4.75)*	9.11 (-1.48,19.69)	-0.05 (-0.08,-0.03)***
Change in level *β*_*2*_	61.84 (21.00,102.68)**	-93.46 (-237.24,50.31)	-1.90 (-2.41,-1.38)***
Change in trend *β*_*3*_	-1.13 (-8.17,5.91)	-13.14 (-29.56,3.28)	0.01 (-0.04,0.06)
Seasonal effects sin *β*_*4*_	-5.16 (-22.97,12.65)	-7.54 (-81.48,66.41)	0.13 (-0.06,0.32)
Seasonal effects cos *β*_*5*_	-1.24 (-18.14,15.67)	4.70 (-48.14,57.53)	-0.16 (-0.34,0.03)
Constant *β*_*0*_	49.86 (26.91,72.81)***	256.79 (144.42,369.17)***	5.21 (4.90,5.53)***
Alternative drugs			
Baseline trend *β*_*1*_	4.86 (0.42,9.31)*	28.90 (3.24,54.55)*	0.02 (0.00,0.04)
Change in level *β*_*2*_	-36.17 (-106.65,34.31)	-211.47 (-606.34,183.40)	-0.09 (-0.38,0.20)
Change in trend *β*_*3*_	7.85 (-2.85,18.55)	41.45 (-17.99,100.88)	-0.02 (-0.06,0.02)
Seasonal effects sin *β*_*4*_	-1.94 (-34.66,30.79)	1.19 (-190.62,193.00)	0.02 (-0.13,0.16)
Seasonal effects cos *β*_*5*_	-5.64 (-30.32,19.04)	-50.78 (-198.45,96.90)	-0.10 (-0.29,0.08)
Constant *β*_*0*_	160.54 (120.04,201.05)***	861.41 (612.60,1110.23)***	5.29 (5.03,5.56)***
Fluoxetine			
Baseline trend *β*_*1*_	1.76 (0.39,3.13)*	13.87 (3.35,24.39)*	0.01 (0.00,0.02)*
Change in level *β*_*2*_	-8.62 (-30.09,12.84)	-79.84 (-242.61,82.93)	-0.24 (-0.40,-0.09)**
Change in trend *β*_*3*_	0.76 (-2.28,3.80)	6.08 (-17.02,29.18)	0.00 (-0.02,0.02)
Seasonal effects sin *β*_*4*_	2.30 (-9.99,14.59)	19.21 (-76.02,114.44)	-0.01 (-0.09,0.07)
Seasonal effects cos *β*_*5*_	-2.53 (-12.31,7.26)	-17.69 (-93.15,57.78)	0.05 (-0.03,0.13)
Constant *β*_*0*_	23.03 (8.53,37.52)**	172.97 (63.25,282.69)**	7.52 (7.38,7.66)***
Sherqulin			
Baseline trend *β*_*1*_	3.10 (-0.43,6.63)	15.02 (-3.14,33.19)	0.00 (-0.01,0.02)
Change in level *β*_*2*_	-27.54 (-85.17,30.09)	-131.64 (-418.25,154.97)	0.00 (-0.17,0.18)
Change in trend *β*_*3*_	7.09 (-1.49,15.67)	35.37 (-6.83,77.57)	0.00 (-0.03,0.02)
Seasonal effects sin *β*_*4*_	-4.24 (-30.46,21.98)	-18.02 (-156.67,120.63)	0.03 (-0.11,0.16)
Seasonal effects cos *β*_*5*_	-3.11 (-24.82,18.59)	-33.09 (-149.40,83.22)	-0.11 (-0.24,0.03)
Constant *β*_*0*_	137.52 (105.35,169.69)***	688.44 (505.60,871.29)***	4.92 (4.68,5.15)***
SSRIs			
Baseline trend *β*_*1*_	10.27 (1.70,18.84)*	65.70 (13.93,117.47)*	0.01 (-0.01,0.02)
Change in level *β*_*2*_	31.17 (-97.66,160.00)	-655.02 (-1384.02,73.97)	-1.56 (-1.95,-1.18)***
Change in trend *β*_*3*_	12.77 (-8.97,34.52)	37.81 (-53.05,128.66)	-0.02 (-0.08,0.04)
Seasonal effects sin *β*_*4*_	-2.94 (-66.56,60.68)	37.88 (-348.48,424.23)	0.06 (-0.14,0.26)
Seasonal effects cos *β*_*5*_	-15.84 (-59.41,27.72)	-116.40 (-355.01,122.21)	-0.15 (-0.31,0.01)
Constant *β*_*0*_	274.68 (200.27,349.09)***	1658.80 (1215.55,2102.05)***	6.05 (5.86,6.24)***

*p < 0.05, **p < 0.01, ***p < 0.001.

The DDDs of policy-related drugs has a baseline trend increased before the “4 + 7” pilot policy by 5.41 thousand (*p* < 0.05). The expenditures on policy-related drugs (*β*_*1*_ = 36.81, *p* < 0.05) represented an increasing baseline trend. And the expenditures on policy-related drugs showed decreasing in level with statistical significance (*β*_*3*_ = -443.55, *p* < 0.05). Moreover, the DDDc of those drugs had a substantial drop of 2.93 CNY (*p* < 0.001) after implementing the policy, but the change in trend after the intervention had only decreased by 0.02 CNY with no statistical significance.

The trend coefficient indicated that DDDs of Escitalopram increased after the implementation of the policy (*β*_*3*_ = 6.06, *p* < 0.05). The expenditures of Escitalopram with level coefficient: *β*_*2*_ = -350.09 (*p*<0.05), on the other hand, decreased. DDDc of Escitalopram showed a positive relation to expenditure, which also dropped by 3.22 CNY(*p* < 0.001). Similarly, DDDs of Paroxetine increased by the influence of the policy with a level coefficient: *β*_*2*_ = 61.84 and *p* < 0.01. DDDc of Paroxetine dropped by 1.90 CNY (*p* < 0.001) after the policy was launched.

The model for alternative drugs suggested that over the period studied, the baseline trend was a 4.86 thousand increase in the DDDs per month (*p* < 0.05). The analysis showed a change in the baseline trend of expenditures of a 28.90 thousand CNY increase. And Fluoxetine had a similar change in the baseline trend.

Overall, for SSRIs, the post-intervention period presented an increase in DDDs, which level coefficient (*β*_*2*_) equaled 31.17, and trend coefficient (*β*_*3*_) equaled 12.77, but the *p*-value both were more than 0.05. The expenditures demonstrated a decreasing trend with no statistical significance (level coefficient: *β*_*2*_ = -655.02, *p* > 0.05). After the intervention, there was a significant decline in DDDc since the level coefficient was equaled to -1.56 (*p* < 0.001).

Table 7 indicated that DDDs of non-winning products significantly decreased as shown in the regression with a level coefficient (*β*_*2*_) of -156.83 (*p* < 0.001) and trend coefficient (*β*_*3*_) of -10.81 (*p* < 0.001). As well as the expenditures on non-winning products they also had a significant decrease with a level coefficient (*β*_*2*_) of -1028.73 (*p* < 0.001) and trend coefficient (*β*_*3*_) of -64.38 (*p* < 0.001). Furthermore, DDDc had an increase of 0.31 CNY (*p* < 0.01) after the implementation of the policy. Table 7 also indicated that DDDs of non-winning products in Escitalopram and Paroxetine significantly decreased as shown in the regression with level coefficient and trend coefficient (*p* < 0.01).

**TABLE 7:** The result of ITS analysis of SSRIs policy-related drugs in Shenzhen.

Categories	DDDs (thousands)	Expenditures (thousands of CNY)	DDDc (CNY)
	Coef. (95%*C.I.*)	Coef. (95%*C.I.*)	Coef. (95%*C.I.*)
Escitalopram			
Non-winning products			
Baseline trend *β*_*1*_	2.94 (0.40,5.48)*	27.94 (8.64,47.24)**	0.04 (-0.06,0.14)
Change in level *β*_*2*_	-71.16 (-116.78,-25.54)**	-688.92 (-1064.63,-313.21)**	-1.03 (-3.09,1.03)
Change in trend *β*_*3*_	-6.48 (-9.86,-3.09)**	-45.89 (-71.07,-20.70)**	0.27 (-0.06,0.59)
Seasonal effects sin *β*_*4*_	6.48 (-13.80,26.76)	54.52 (-116.80,225.83)	-0.04 (-0.71,0.64)
Seasonal effects cos *β*_*5*_	-1.79 (-12.47,8.90)	-38.58 (-135.54,58.38)	-0.23 (-0.66,0.20)
Constant *β*_*0*_	63.93 (43.82,84.04)***	539.04 (401.61,676.47)***	8.51 (7.46,9.56)***
Winning products			
Baseline trend *β*_*1*_	-0.05 (-0.24,0.13)	-0.24 (-1.07,0.59)	-
Change in level *β*_*2*_	76.66 (48.13,105.19)***	338.83 (212.72,464.95)***	-
Change in trend *β*_*3*_	12.53 (7.17,17.90)***	55.39 (31.68,79.10)***	-
Seasonal effects sin *β*_*4*_	-2.33 (-10.47,5.81)	-10.30 (-46.28,25.68)	-
Seasonal effects cos *β*_*5*_	-7.18 (-17.51,3.15)	-31.74 (-77.38,13.91)	-
Constant *β*_*0*_	0.35 (-1.73,2.43)	1.55 (-7.63,10.74)	-
Paroxetine			
Non-winning products			
Baseline trend *β*_*1*_	2.58 (0.40,4.75)*	9.19 (-1.33,19.71)	-0.05 (-0.08,-0.03)***
Change in level *β*_*2*_	-85.68 (-117.68,-53.68)***	-339.81 (-494.66,-184.96)***	2.12 (1.40,2.84)***
Change in trend *β*_*3*_	-4.33 (-6.77,-1.90)**	-18.49 (-30.28,-6.71)**	0.17 (0.04,0.30)*
Seasonal effects sin *β*_*4*_	-0.68 (-14.87,13.51)	-0.06 (-71.22,71.10)	0.12 (-0.07,0.30)
Seasonal effects cos *β*_*5*_	6.04 (-5.20,17.28)	16.85 (-30.40,64.09)	-0.20 (-0.40,0.00)
Constant *β*_*0*_	49.61 (26.82,72.39)***	256.37 (144.09,368.65)***	5.22 (4.90,5.53)***
Winning products			
Baseline trend *β*_*1*_	-0.05 (-0.26,0.16)	-0.09 (-0.44,0.26)	-
Change in level *β*_*2*_	147.51 (108.10,186.93)***	246.35 (180.52,312.18)***	-
Change in trend *β*_*3*_	3.20 (-3.24,9.65)	5.35 (-5.41,16.11)	-
Seasonal effects sin *β*_*4*_	-4.48 (-14.48,5.53)	-7.48 (-24.19,9.23)	-
Seasonal effects cos *β*_*5*_	-7.28 (-18.53,3.98)	-12.15 (-30.94,6.64)	-
Constant *β*_*0*_	0.25 (-1.84,2.35)	0.42 (-3.07,3.92)	-
**Policy-related drugs**			
Non-winning products			
Baseline trend *β*_*1*_	5.51 (1.02,10.00)*	37.13 (9.17,65.10)*	-0.01 (-0.07,0.05)
Change in level *β*_*2*_	-156.83 (-231.64,-82.03)***	-1028.73 (-1540.24,-517.23)***	0.67 (-0.49,1.84)
Change in trend *β*_*3*_	-10.81 (-16.13,-5.49)***	-64.38 (-97.73,-31.03)**	0.31 (0.12,0.49)**
Seasonal effects sin *β*_*4*_	5.80 (-26.60,38.20)	54.46 (-171.12,280.04)	0.24 (-0.22,0.69)
Seasonal effects cos *β*_*5*_	4.25 (-15.84,24.35)	-21.73 (-150.34,106.87)	-0.39 (-0.74,-0.04)*
Constant *β*_*0*_	113.53 (74.44,152.63)***	795.41 (574.52,1016.30)***	7.16 (6.44,7.89)***
Winning products			
Baseline trend *β*_*1*_	-0.11 (-0.49,0.28)	-0.33 (-1.48,0.83)	-
Change in level *β*_*2*_	224.17 (165.38,282.96)***	585.18 (417.04,753.33)***	-
Change in trend *β*_*3*_	15.74 (5.20,26.27)**	60.74 (29.47,92.01)***	-
Seasonal effects sin *β*_*4*_	-6.81 (-22.65,9.04)	-17.77 (-64.67,29.12)	-
Seasonal effects cos *β*_*5*_	-14.46 (-35.27,6.36)	-43.89 (-106.44,18.66)	-
Constant *β*_*0*_	0.60 (-3.47,4.68)	1.98 (-10.48,14.43)	-

*p < 0.05, **p < 0.01, ***p < 0.001.

Table 7 indicated that DDDs of winning products significantly increased as shown in the regression with a level coefficient (*β*_*2*_) of -224.17 (*p* < 0.001) and trend coefficient (*β*_*3*_) of 15.74 (*p* < 0.001). As well to the expenditures of winning products they also had a significant increase with a level coefficient (*β*_*2*_) of 585.18 (*p* < 0.001) and trend coefficient (*β*_*3*_) of 60.74 (*p* < 0.001). The winning products in Escitalopram and Paroxetine The winning products in Escitalopram showed similar changes. The winning products in Paroxetine only showed an increase in level.

Table 8 demonstrated that both DDDs and expenditures on branded products showed a significant decrease in the regression in the post-intervention period (level coefficient of DDDs: *β*_*2*_ = -57.65, *p* < 0.01, trend coefficient of DDDs: *β*_*3*_ = -3.44, *p* < 0.01; level coefficient of expenditure: *β*_*2*_ = -712.98, *p* < 0.01, trend coefficient of expenditure: *β*_*3*_ = -40.10, *p* < 0.01). The DDD of branded products shrunk as shown in Table 8 (level coefficient: *β*_*2*_ = -1.47, *p* < 0.001). The branded drugs in Escitalopram and Paroxetine have similar changes.

**TABLE 8:** The result of ITS analysis of SSRIs policy-related drugs in Shenzhen.

Categories	DDDs (thousands)	Expenditures (thousands of CNY)	DDDc (CNY)
	Coef. (95%*C.I.*)	Coef. (95%*C.I.*)	Coef. (95%*C.I.*)
Escitalopram			
Branded drugs			
Baseline trend *β*_*1*_	2.11 (1.03,3.19)***	27.27 (13.31,41.23)***	0.00 (0.00,0.00)
Change in level *β*_*2*_	-43.99 (-66.42,-21.56)***	-592.76 (-882.19,-303.33)***	-1.07 (-1.39,-0.74)***
Change in trend *β*_*3*_	-2.35 (-4.03,-0.68)**	-31.50 (-52.48,-10.53)**	-0.04 (-0.09,0.02)
Seasonal effects sin *β*_*4*_	3.63 (-6.99,14.25)	42.58 (-92.58,177.73)	0.02 (-0.02,0.06)
Seasonal effects cos *β*_*5*_	-4.69 (-11.64,2.26)	-55.53 (-140.85,29.80)	0.03 (-0.03,0.08)
Constant *β*_*0*_	26.73 (18.38,35.07)***	343.70 (236.26,451.14)***	12.88 (12.87,12.89)***
Generic drugs			
Baseline trend *β*_*1*_	0.77 (-0.85,2.38)	0.43 (-6.11,6.96)	-0.09 (-0.18,-0.01)*
Change in level *β*_*2*_	49.49 (22.91,76.07)***	242.67 (122.95,362.39)***	0.77 (0.05,1.49)*
Change in trend *β*_*3*_	8.41 (2.61,14.21)**	41.01 (15.74,66.27)**	0.10 (0.02,0.19)*
Seasonal effects sin *β*_*4*_	0.52 (-13.63,14.68)	1.65 (-58.31,61.60)	0.01 (-0.14,0.15)
Seasonal effects cos *β*_*5*_	-4.27 (-16.59,8.04)	-14.79 (-69.58,39.99)	0.11 (-0.13,0.34)
Constant *β*_*0*_	37.55 (24.34,50.77)***	196.89 (147.76,246.02)***	5.49 (4.28,6.70)***
Paroxetine			
Branded drugs			
Baseline trend *β*_*1*_	0.26 (-0.54,1.07)	2.14 (-4.38,8.67)	0.00 (0.00,0.00)
Change in level *β*_*2*_	-13.66 (-24.97,-2.34)*	-120.22 (-213.48,-26.96)*	-0.79 (-1.24,-0.34)***
Change in trend *β*_*3*_	-1.09 (-2.07,-0.11)*	-8.60 (-16.17,-1.03)*	-0.05 (-0.12,0.02)
Seasonal effects sin *β*_*4*_	0.28 (-5.42,5.98)	2.88 (-42.99,48.75)	0.03 (-0.03,0.08)
Seasonal effects cos *β*_*5*_	-0.56 (-3.68,2.57)	-3.26 (-28.28,21.76)	0.04 (-0.04,0.12)
Constant *β*_*0*_	20.90 (11.82,29.98)***	168.85 (95.50,242.20)***	8.08 (8.07,8.09)***
Generic drugs			
Baseline trend *β*_*1*_	2.26 (0.75,3.77)**	6.96 (2.43,11.49)**	0.00 (0.00,0.00)
Change in level *β*_*2*_	75.49 (37.88,113.11)***	26.76 (-47.82,101.33)	-1.31 (-1.40,-1.22)**
Change in trend *β*_*3*_	-0.04 (-6.73,6.65)	-4.54 (-16.53,7.44)	-0.01 (-0.03,0.00)
Seasonal effects sin *β*_*4*_	-5.44 (-19.03,8.15)	-10.41 (-42.63,21.80)	0.00 (-0.01,0.01)
Seasonal effects cos *β*_*5*_	-0.68 (-15.88,14.52)	7.96 (-27.11,43.02)	0.01 (-0.01,0.02)
Constant *β*_*0*_	28.96 (13.52,44.40)**	87.95 (41.34,134.55)**	3.05 (3.05,3.05)***
Policy-related drugs			
Branded drugs			
Baseline trend *β*_*1*_	2.38 (0.64,4.11)**	29.42 (10.38,48.46)**	0.04 (0.00,0.07)*
Change in level *β*_*2*_	-57.65 (-89.51,-25.79)**	-712.98 (-1079.18,-346.77)***	-1.47 (-2.02,-0.93)***
Change in trend *β*_*3*_	-3.44 (-5.65,-1.24)**	-40.10 (-65.04,-15.16)**	-0.01 (-0.14,0.12)
Seasonal effects sin *β*_*4*_	3.91 (-10.74,18.56)	45.45 (-120.88,211.79)	0.14 (-0.23,0.51)
Seasonal effects cos *β*_*5*_	-5.25 (-13.88,3.38)	-58.79 (-156.41,38.84)	-0.07 (-0.30,0.17)
Constant *β*_*0*_	47.63 (32.04,63.21)***	512.55 (350.19,674.91)***	10.89 (10.51,11.26)***
Generic drugs			
Baseline trend *β*_*1*_	3.03 (0.10,5.96)*	7.39 (-2.64,17.42)	-0.07 (-0.12,-0.01)*
Change in level *β*_*2*_	124.99 (66.06,183.92)***	269.43 (98.45,440.41)**	-0.29 (-0.76,0.18)
Change in trend *β*_*3*_	8.37 (-3.54,20.27)	36.46 (1.19,71.74)*	0.11 (0.04,0.17)**
Seasonal effects sin *β*_*4*_	-4.92 (-31.06,21.22)	-8.77 (-96.51,78.98)	0.08 (-0.05,0.22)
Seasonal effects cos *β*_*5*_	-4.95 (-31.30,21.39)	-6.83 (-89.97,76.30)	-0.01 (-0.22,0.21)
Constant *β*_*0*_	66.51 (41.14,91.89)***	284.84 (208.37,361.31)***	4.48 (3.69,5.28)***

*p < 0.05, **p < 0.01, ***p < 0.001.

Table 8 demonstrated that both DDDs and expenditures of generic products showed a significant increase in the regression in the post-intervention period (level coefficient of DDDs: *β*_*2*_ = 124.99, *p* < 0.001; level coefficient of expenditure: *β*_*2*_ = 269.43, *p* < 0.01, trend coefficient of expenditure: *β*_*3*_ = -40.10, *p* < 0.01). But the DDDc of generic products increased as shown in Table 8 (trend coefficient: *β*_*3*_ = 0.11, *p* < 0.01). The generic drugs in Escitalopram have similar changes.

## Discussion

The “4 + 7” pilot policy has shown the initial success of lowering prices by government-oriented group purchases. Thus, this study analyzed the effect of the “4 + 7” pilot policy on the daily cost of SSRIs in Shenzhen. For example, the DDD of SSRIs decreased with the “4 + 7” pilot policy by 1.56 yuan (*p* < 0.001). The DDD of policy-related drugs had an immediate drop of 2.93 yuan (95%*CI* -3.68 to -2.19, *p* < 0.001) under the “4 + 7” pilot policy. The “4 + 7” pilot policy was designed to achieve lower prices through competitive bidding processes between accredited generic drug manufacturers.

The reduced price of drugs may improve the accessibility of drugs. A previous survey revealed that the proportion of medication costs in outpatient costs was 74.01%, conducted in five cities (Beijing, Changsha, Chengdu, Shanghai, and Suzhou) (Hu et al., 2007). The result revealed that the proportion of medication costs in outpatient costs was 86.14%, which surveyed 652 outpatients with depression in Shanghai (Zhou Xuedong et al., 2008). Reducing the burden of drug costs may improve the compliance of depressive disorder patients. Medicine’s price control measures were used to increase medicine’s affordability (Rawson, 2020). This study revealed that the “4 + 7” pilot policy led to an increase in the total volume of SSRIs, as well as each of the four study medications. The DDDs of SSRIs increased by 49.85%. The result has shown a 76.70% increase in the purchased volume of policy-related drugs and a 3.39% decrease in the expenditure on policy-related drugs. Over the first 9 months of implementation, this study found that the “4 + 7” pilot policy increased the proportion of purchasing policy-related drugs. It was consistent with the result of the study conducted in one of 11 pilot cities Dalian (Sheng Liang-Liang and Hu, 2019).

New Zealand controlled pharmaceutical expenditures by a combination of strong negotiation, bundling agreements, tendering sole supply, and contracts. Then it resulted in immediate savings on pharmaceutical expenditures with up to 90 percent on some drugs, despite a 50% increase in volumes (Lybecker, 2013). After the implementation of the “4 + 7” pilot policy, policy-related drugs decreased by 443.55 thousand CNY. The “4 + 7” pilot policy led to significant savings and improvement in the efficient resource allocation of the healthcare system, which was consistent with previous studies (Qi et al., 2020; Yang et al., 2021).

But the policy effects were smaller observed in SSRIs, including price reductions, cost-saving, unleashing medication demand, and improving accessibility. A previous study revealed that the largest reduction in spending occurred on drugs for the treatment of cardiovascular diseases in the “4 + 7” pilot policy (Chen et al., 2021). Another study in Shenzhen revealed that the post-intervention period witnessed a significant increase in the regression level for nucleos(t)ide analogs DDDs (level coefficient: *β*_*2*_ = 631.87, *p* < 0.05). The expenditures (trend coefficient: *β*_*3*_ = 392.24, *p* < 0.05) and DDDc (level coefficient: *β*_*2*_ = −6.17, *p* < 0.001; trend coefficient: *β*_*3*_ = −0.21, *p* < 0.05) of NAs showed decreasing trend in the post-intervention period (Wen et al., 2021). It may be due to SSRIs having less market competition because of fewer generic drug manufacturers, as well as less willingness for healthcare providers to clinical conversion in patients taking antidepressant medication for a long time (Yang et al., 2021). The volume and expenditures of alternative drugs increased after the “4 + 7” pilot policy. It was a side effect of pharmaceutical policies (Kwon et al., 2013; Kwon et al., 2019; Chen et al., 2020; Yang et al., 2021). In this study, the volume and expenditures of alternative drugs didn’t show statistic significant changes in the interrupted time-series analysis. Most of depression patients got prescription based on a doctor’s diagnosis. We need monitor the using of SSRIs for a long term to ensure rational use of drugs.

The volume-based procurement policy is aimed at reducing pharmaceutical expenditures by creating economies of scale and improving purchasing power (Seidman and Atun, 2017). On the one hand, pharmaceutical companies offered lower prices in exchange for a larger volume of purchases, given the result of winning drugs replacing the non-winning drugs. Winning products were given priority to use, which resulted in putting winning products in the place of non-winning products (Jialing et al., 2021). This study found that the “4 + 7” pilot policy increased the proportion of purchasing winning drugs, with an increment of 85.60 percent. The volume of non-winning products had a significant decrease (*β*_*2*_ = -156.83, *p* < 0.001; *β*_*3*_ = -10.81, *p* < 0.001). The volume of non-winning products experienced attenuation following the entry of winning products, and both Escitalopram and Paroxetine decreased. Because all public medical institutions (including public hospitals and government-run primary healthcare centers) in the “4 + 7″ pilot cities need to give priority to using drugs which won the bidding.

Volume-based procurement policy potentially reshaped the market share of pharmaceuticals by substituting branded with generic drugs (World Health Organization, 2007; Waning et al., 2009; Lybecker, 2013). The proportion of branded drugs decreased by 31.55 percent. Only one company would win the bidding for each policy-related drug in the “4 + 7” pilot policy. And the company won the bidding in the “4 + 7” pilot policy for Escitalopram and Paroxetine both were generic products. All public hospitals and primary healthcare centers in the “4 + 7″ pilot cities gave priority to using drugs that won the bidding. In this way, the decrease mainly occurred in branded drugs. The promotion of generic drugs using was a commonly used strategy, which could improve medicine’s affordability and accessibility. Most of the Association of Southeast Asian Nations (ASEAN) countries also applied generic medicine promotion, which can enhance the use of much cheaper generic medicines (You et al., 2019). The result revealed that the volume of branded drugs both showed a significant decrease in the regression level and trend in the post-intervention period (*β*_*2*_ = -57.65, *p* < 0.01; *β*_*3*_ = -3.44, *p* < 0.01). The volume-based procurement policy accredited generics in place of off-patent branded drugs, which also resulted in lower SSRIs total drug purchasing costs. It was consistent with previous studies (Dylst et al., 2015; Wouters et al., 2017). The volume-based procurement policy relieves the overall drug burden on patients (Son, 2021). It also accomplished the goal of controlling drug costs (Nunes et al., 2020). For example, generic substitution was compulsory in Greece (Wouters et al., 2017). Policymakers usually require generic prescribing and substitution to achieve significant savings in the United States. They also streamline the generic drug approval process for this purpose.

The implementation of the “4 + 7” pilot policy, improves the quality of medicines because the generic drugs winning the bid got generic quality consistency evaluation approval (Lijun, 2019). Generic drugs which did not get generic quality consistency evaluation approval would be out of the market very soon. Some small pharmaceutical companies could not take part in the bidding or lost the bid. Then they may stop manufacturing and exit the market (Hu et al., 2015). The volume-based procurement policy drove small drug manufacturers with inferior research and production capacity out of business.

However, it is unclear whether the lowest price for a drug will always be the best value, and it is an issue that many purchasers must consider (van Valen et al., 2018). In the “4 + 7” pilot policy, sole supply may cause drug shortages (Zhang, 2019). The researchers also found that later rounds of volume-based procurement have to change the number of pharmaceuticals in the bidding rules. Considering all other factors will lead to the timely, reliable delivery of safe, high-quality products, and ultimately result in lower prices from increased competition. Big data analytics might help set reasonable cap prices and monitor the real-world data and evidence to support price negotiations for procurement. To deliver safe and cost-effective medicines, it was necessary to systematically evaluate the effectiveness, safety and economics are necessary (Li et al., 2018). With the moving toward value-based medical, health decision-making pays more attention to Health Technology Assessment. In volume-based procurement. Health Technology Assessment could serve as an effective tool based on real-world data.

More rounds of volume-based procurement have been rapidly carried out in the country, and assessing long-term trends in volume and expenditure is significant. Evaluation of the effect of policy could guide policymakers, healthcare providers, and patients to better understand the reform and adapt accordingly. And it is still important to evaluate the policy effects on special disease categories by assessing further data from more rounds of volume-based procurements.

The main strength of this study was using ITS quantitative analysis of the impact of the “4 + 7″ pilot policy. It may be a valuable reference for policy effect evaluation. It offered suggestions for policy promotion.

## Limitations

The present study had some limitations that should be borne in mind when interpreting the results. First, one of the limitations was the lack of inclusion of drugstores, as one of the main stakeholders of the pharmaceutical industry in the study. The reason was only public medical institutions were included in the purchasing alliance in the “4 + 7″ pilot policy. Second, this study only used 9 months of time series data post-intervention. In exploring the long-term trend of the “4 + 7″ pilot policy, it would be better if this study could get access to all pilot cities as research objects and full purchasing cycle as research time points. The purchasing cycle was 12 months. The drug will be purchased with a bidding price until the purchase cycle expired. We didn’t get purchasing records for the other 3 months during purchasing cycle at present. But the trend would same as the result of this study.

## Conclusion

The volume-based procurement has successfully led to price reductions and improved the affordability of medicines, especially for those with chronic diseases. The volume-based procurement has demonstrated initial success in reshaping the composition of the Chinese pharmaceutical market in favor of generics with high quality and low prices. Future studies are needed to investigate the long-term impact of the volume-based procurement policy on various outcomes, such as patient outcomes, drug utilization, and changes in the pharmaceutical industry.

## Data Availability

The original contributions presented in the study are included in the article/Supplementary Material further inquiries can be directed to the corresponding authors.

## Abbreviations

CNY, Chinese yuan renminbi; OECD, Organization for Economic Co-operation and Development; DDD, Defined Daily Dose DDDc, Defined Daily Dose cost; ITS, Interrupted time-series.
