# Supplementary figures and images for: The impacts of the “4+7” pilot policy on the volume, expenditures, and daily cost of Serotonin-Specific Reuptake Inhibitors (SSRIs) antidepressants: A quasi-experimental study
Source: Front Pharmacol. 2022 Aug 17;13:829660. doi: 10.3389/fphar.2022.829660 (PMC9428282; doi:10.3389/fphar.2022.829660)

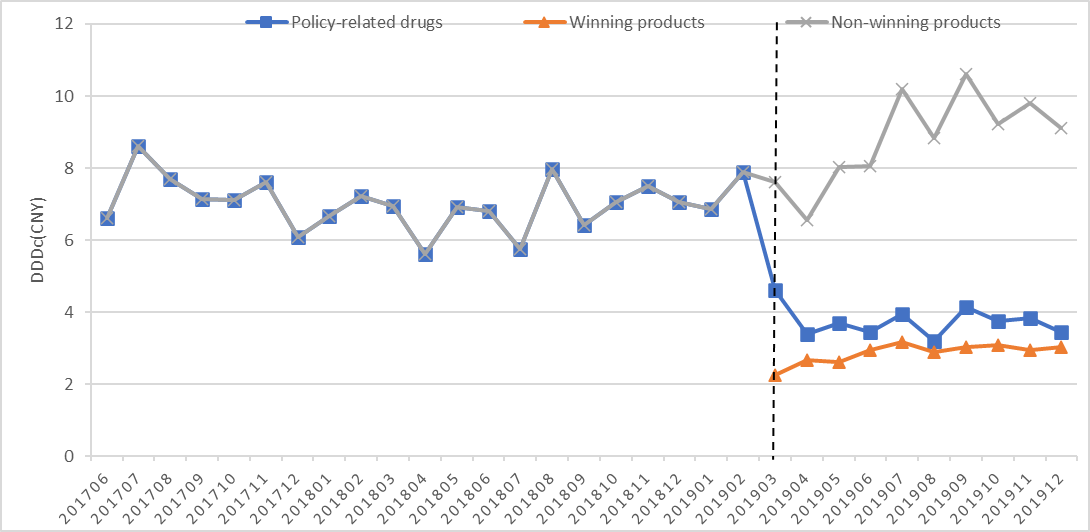

Supplement: Supplementary file 1 [file Image6.TIF]

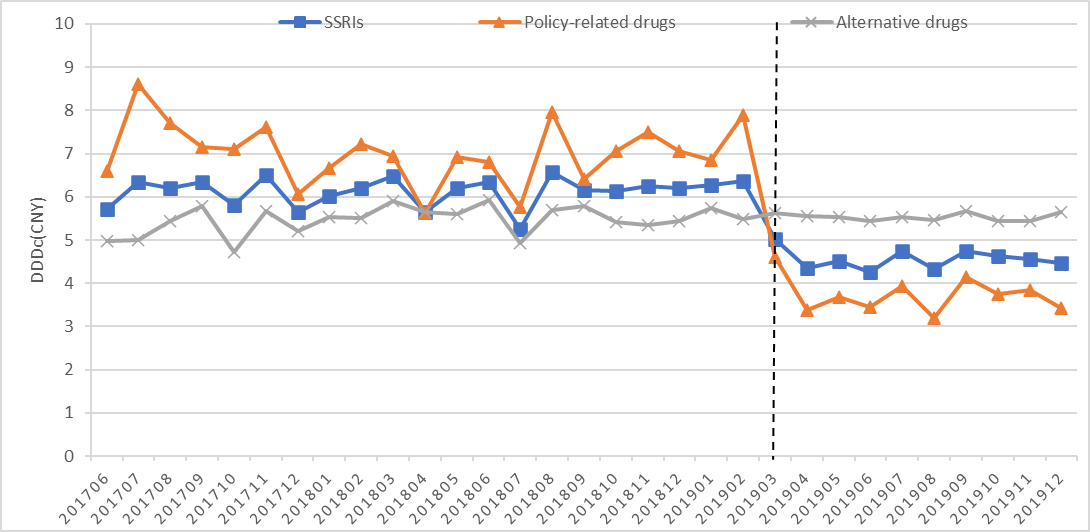

Supplement: Supplementary file 2 [file Image3.TIF]

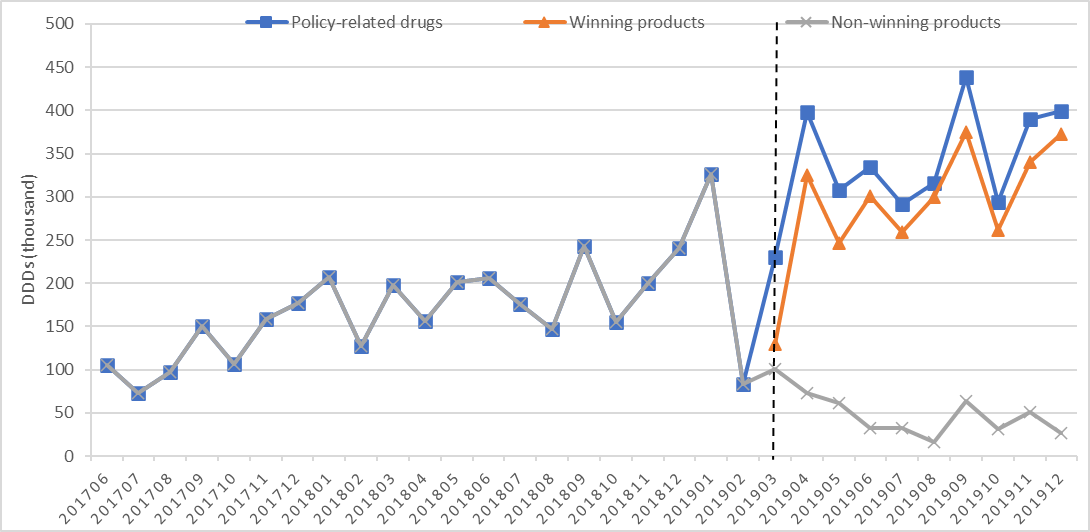

Supplement: Supplementary file 3 [file Image4.TIF]

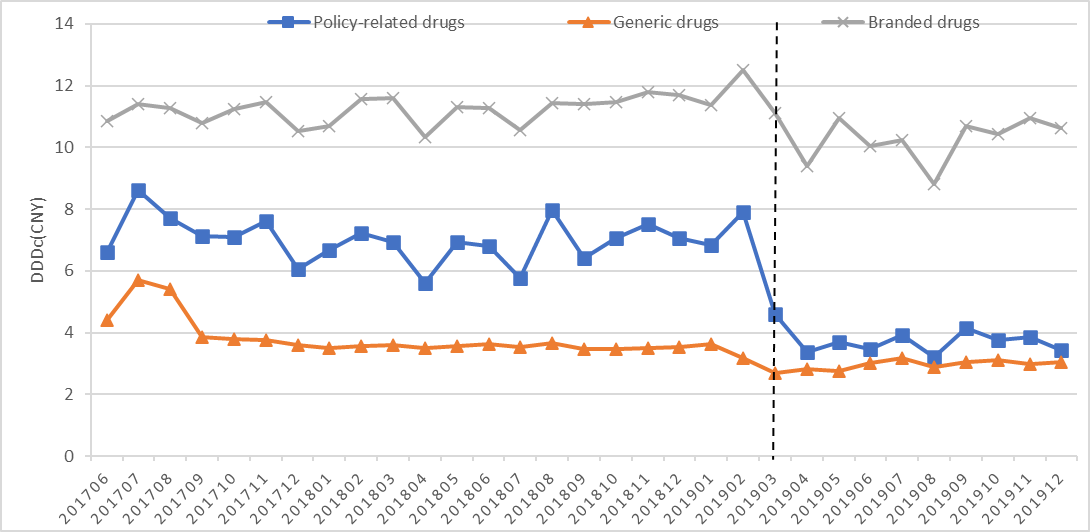

Supplement: Supplementary file 4 [file Image9.TIF]

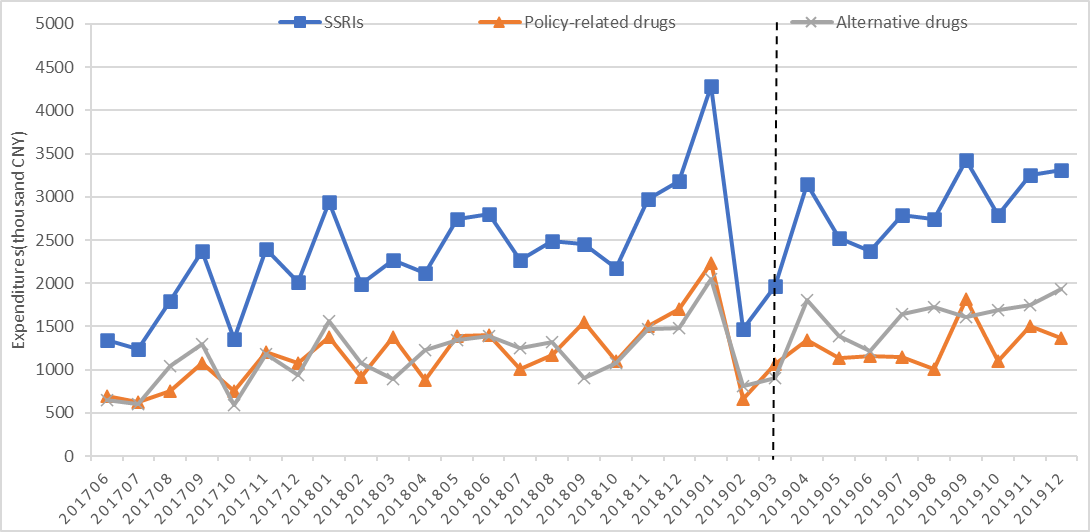

Supplement: Supplementary file 5 [file Image2.TIF]

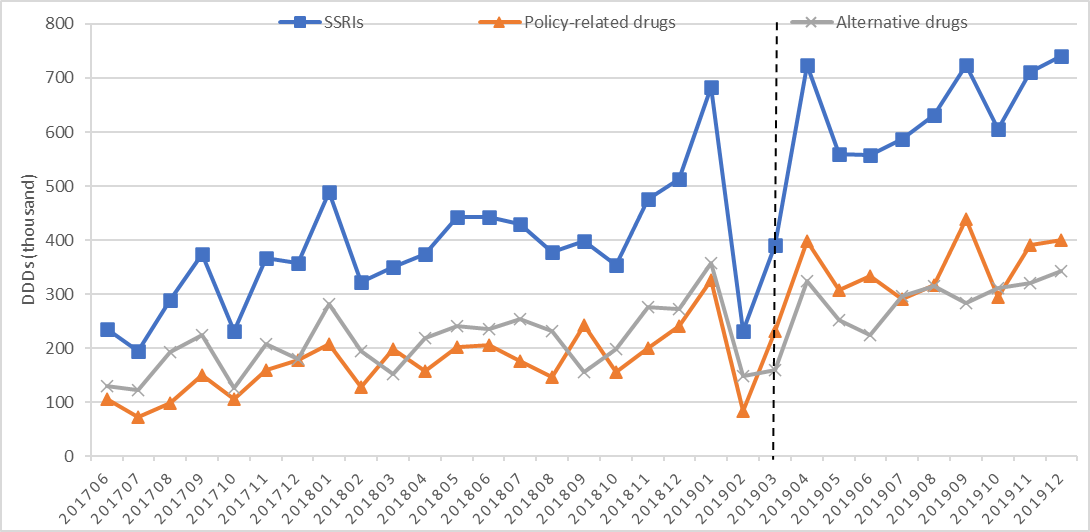

Supplement: Supplementary file 6 [file Image1.TIF]

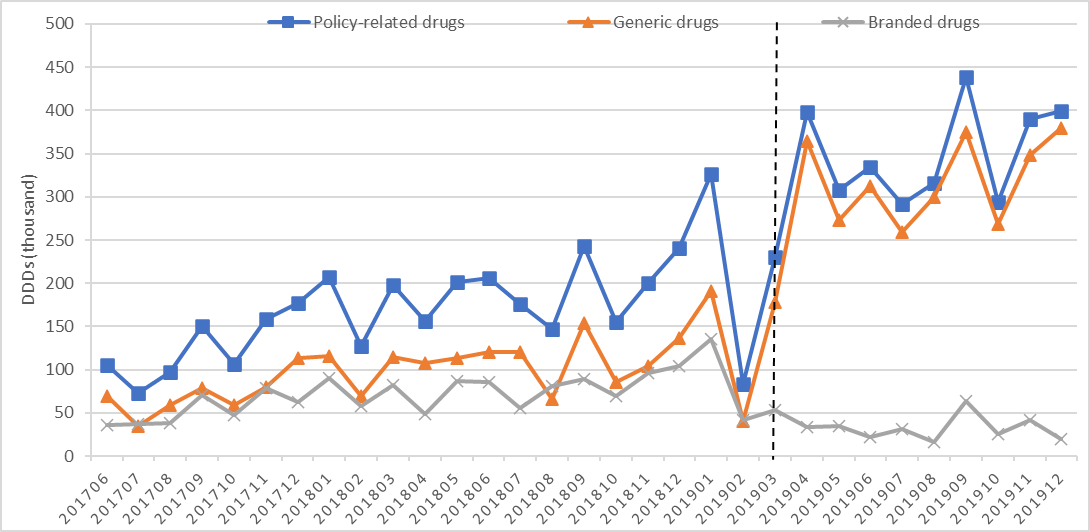

Supplement: Supplementary file 7 [file Image7.TIF]

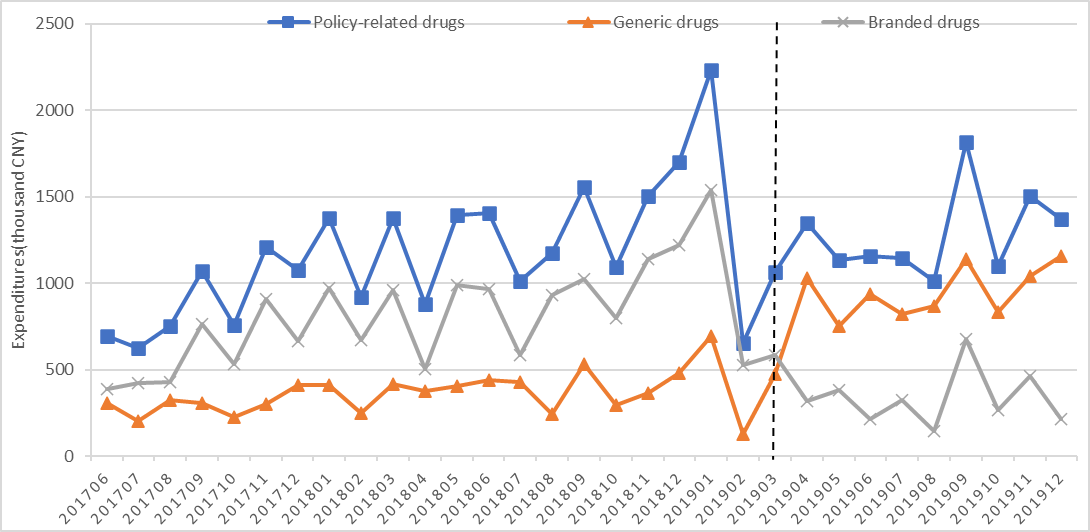

Supplement: Supplementary file 8 [file Image8.TIF]

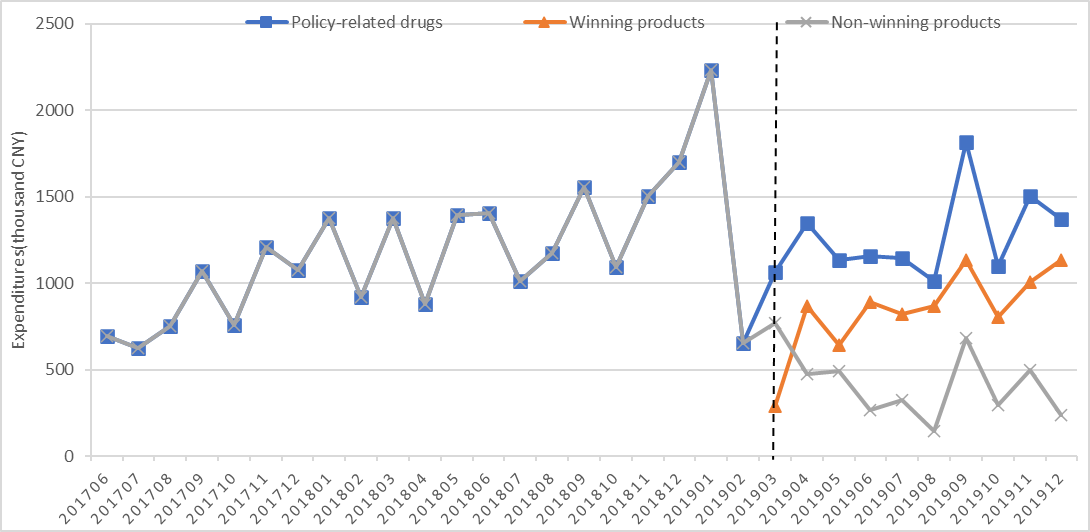

Supplement: Supplementary file 9 [file Image5.TIF]
